# Hip reconstruction is more painful than spine fusion in children with cerebral palsy

**DOI:** 10.1007/s11832-015-0656-x

**Published:** 2015-05-06

**Authors:** M. Wade Shrader, John Jones, Mandy N. Falk, Greg R. White, David R. Burk, Lee S. Segal

**Affiliations:** Division of Pediatric Orthopaedic Surgery, Phoenix Children’s Hospital, 1919 East Thomas Road, Phoenix, AZ 85016 USA

**Keywords:** Cerebral palsy, Hip reconstruction, Hip dysplasia, Scoliosis, Spine fusion, Pain control, Pain assessment

## Abstract

**Purpose:**

Concerns about pain control in patients with cerebral palsy (CP) are especially anxiety provoking for parents, given the fact that spasticity, communication issues, and postoperative muscle spasms are significant problems that make pain control difficult in these patients. A better understanding of the magnitude and quality of the pain these patients experience after our surgical procedures would better prepare the patients and their families. The purpose of this study is to quantify the amount of postoperative pain in children with CP undergoing hip reconstruction and spinal fusion. Specifically, the study will compare pain scores and the amount of narcotics used between the two groups.

**Materials and methods:**

This is a retrospective chart review of a consecutive series of children with CP (GMFCS levels IV and V) over a 5-year period undergoing hip reconstruction (femoral osteotomy, pelvic osteotomy, or both) and posterior spinal fusion (PSF) at a tertiary-care pediatric hospital. The primary end point was the total opioid used by the patient during the hospitalization, by converting all forms of narcotics to morphine equivalents. The secondary end point was the documentation of pain with standard pain scores at standard time points postoperatively. Adverse effects related to pain management were documented for both groups. Student’s *t*-tests were utilized to statistically compare differences between the groups, with significance determined at *p* < 0.05.

**Results:**

Forty-two patients with CP who underwent hip reconstruction (mean age 8.8 years) were compared to 26 patients who underwent PSF (mean age 15.4 years). The total opioid used, normalized by body weight and by days length of stay (DLOS), in the hip group was 0.49 mg morphine/kg/DLOS, compared to 0.24 for the spine group (*p* = 0.014). The mean pain score for the hip group was 1.52, compared to 0.72 for the spine group (*p* = 0.013). There were no significant differences in the occurrence of adverse effects related to pain management between the two groups.

**Conclusion:**

Patients with CP undergoing hip reconstruction surgery had significantly more pain, as exhibited by requiring more narcotics and having higher pain scores, than those patients undergoing PSF. The knowledge that hip reconstruction is more painful than PSF for patients with CP will better prepare families about what to expect in the postoperative period and will alert providers to supply better postoperative pain control in these patients.

**Level of evidence:**

III (case control series).

## Introduction

The assessment, impact, and treatment of pain in patients with cerebral palsy (CP), especially those with cognitive impairment and developmental delay, can be difficult, and is an important concern for the practitioner, the family, and the patient [1]. Concerns about postoperative pain control in patients with CP are especially anxiety provoking, given the fact that spasticity, communication issues, and postoperative muscle spasms are significant issues that make pain control difficult in these patients [2–4].

More attention in the literature is being focused on the presence of pain in patients with CP [5, 6]. However, most of the recent reports have documented the high likelihood of chronic pain in adolescents and adults with CP [7–9]. There has been very little investigation on the prevalence, assessment, and control of acute, postoperative pain in these disabled patients [10].

Patients with CP often need a variety of orthopedic surgical interventions to improve their function, provide pain relief, or prevent deterioration of the musculoskeletal system that could negatively impact their future quality of life (QoL) [11–13]. Frequently, patients with CP, especially those with GMFCS levels IV and V, have progressive neuromuscular hip dysplasia, requiring them to undergo hip reconstruction (femoral osteotomy, pelvic osteotomy, and/or both). Similarly, these same patients often develop neuromuscular scoliosis, requiring posterior spinal fusion (PSF).

The concept of an interactive, mutual informed consent process is now the mainstay in our relationships with our patients. Part of the informed consent process is a description of the risks, benefits, and alternatives of the proposed surgical procedure [14]. A discussion of the postoperative period, including how much pain their child may experience, is an expectation of parents [15, 16]. A better understanding of the magnitude and quality of the pain these patients experience after our surgical procedures would better prepare the patients and their families [17–20]. Furthermore, a better understanding of what to expect postoperatively frequently leads to better patient satisfaction ratings [21–23].

The purpose of this study is to quantify the amount of postoperative pain in children with CP undergoing hip reconstruction and PSF. Specifically, the study will compare pain scores and the amount of narcotics (opioid) used between the two groups. The goal of the study is to provide better objective information on postoperative pain in children with CP, so that providers can better prepare their patients and families.

## Materials and methods

This is a retrospective chart review of a consecutive series of children with CP over a 5-year period at a tertiary-care pediatric hospital. Inclusion criteria included patients with diagnosis (ICD-9) codes for CP, with a GMFCS level IV or V, who underwent procedures for hip dysplasia with reconstruction (femoral osteotomy, pelvic osteotomy, or both) and PSF identified by CPT codes for those procedures. An additional inclusion criterion was that the medical records of all patients had to have documented complete pain assessment scores during the first 3 days postoperatively. Exclusion criteria were any child with CP who did not undergo those specific procedures, or those patients who underwent the procedures without a specific diagnosis of CP. Also, any patients who had an anterior spinal release and/or fusion as part of their spine treatment were also excluded from the study. The study was reviewed and approved by the hospital’s Institutional Review Board (IRB).

All of the patients in the hip group underwent soft tissue lengthening with adductor longus and gracilis tenotomy with a fractional lengthening of adductor brevis, and varus derotational osteotomies (VDRO). The VDROs were performed through a standard lateral approach with a supine position and all had a 2-cm femoral shortening performed as a standard portion of the hip reconstruction to decrease soft-tissue tension. Patients older than 7 years of age underwent concomitant pelvic osteotomies with the VDRO if there were any signs of acetabular dysplasia. All pelvic osteotomies were performed with an anterior approach, elevating the gluteal muscles off the outer table of the pelvis; the inner table was not violated. The osteotomies were lateral-based San Diego-type procedures, with the graft being taken from the femoral shortening.

The primary end point was the total opioid used by the patient during the hospitalization, by converting all forms of narcotics (intravenous and oral) to morphine (MSO_4_) equivalents. A normalized opioid value was then obtained by dividing the total opioid used by the weight of the child (in kg) and the number of days of hospitalization (nTOU) (units of mg MSO_4_/kg-days). All of these patients remained on the inpatient unit for at least 3 days postoperatively. The data from these two groups were then analyzed to determine differences in TOU.

The secondary end point was the documentation of pain with standard pain scores at standard time points postoperatively within the first three postoperative days. The pain scores were assessed every 4 h for the first three postoperative days. Three days were chosen as a time point of convenience, since some hip patients are discharged on postoperative day 3. Two pain assessment tools were utilized for patients included in the study. These are both standard pain assessment tools used at our institution. Older patients able to communicate verbally used the visual analog scale (VAS). For non-verbal children, the revised face, legs, activity, cry, and consolability (FLACC) behavioral tool was used [24, 25].

All patients received standard pain control postoperatively through on-demand intravenous narcotics and/or a patient-controlled analgesia (PCA) pump. The standard on-demand intravenous morphine dose at our institution is 0.05 mg/kg every 1 h; the standard morphine PCA dose is 0.02 mg/kg every 10 min, with a 1-h maximum of 0.1 mg/kg. However, occasionally, a substitute opioid with equivalent dosing was used. All of the patients in the hip group also received indwelling epidural catheters (with local anesthetic) for the first 48 h postoperatively.

In addition, all patients in both groups also received scheduled intravenous and oral diazepam (0.1 mg/kg every 6 h) and scheduled intravenous ketorolac (0.5 mg/kg every 6 h, maximum daily dose 60 mg) to assist with adjunctive pain control. These adjunctive pain control modalities were used in both groups, with standardized dosing regiments. In these cohorts of patients, none of the spine group had any additional adjunctive pain control methods, such as intrathecal injections or submuscular pain pumps.

The postoperative protocols for each group were similar, with mobilization out of bed to their wheelchairs either as soon as possible, typically on postoperative days 1 or 2. Postoperative immobilization for the hip group was either with spica casts or with full-time hip abduction pillows and knee immobilizers (Table 1). All patients were discharged with adequate pain control with oral medications, and all patients tolerated transfers from bed to wheelchair. Immobilization for the hip group (either with spica cast or with the hip abduction pillow) was continued full-time for 6 weeks. Physical therapy was not restarted until 6 weeks postoperatively in both groups.

**Table 1:** Patient demographics

	Hip	Spine
Males/females	17 males/25 females	16 males/10 females
Mean age (years)	8.8 (range 4–21)	15.4 (range 10–22)
Length of stay (days)	4.0 (range 3–7)	5.28 (range 3–12)
Deformity (hip—Reimer’s migration %; spine—Cobb angle)	68 % uncovered (range 45–100)	78° (range 50–120)
Immobilization	18 spica cast/22 hip abduction pillow and knee immobilizer	n/a (no TLSO were used)

Adverse effects related to pain management were documented for both groups. Student’s *t*-tests were utilized to statistically compare differences in the normalized TOU and the pain scores between the groups, with significance determined at *p* < 0.05.

## Results

Our institution’s database search identified a consecutive series of 68 patients with CP either undergoing hip reconstruction or PSF during the time period of the study who met the full inclusion criteria for the study. Eight patients were excluded from the study. Five patients in the hip group were excluded, two due to incomplete medical records and three for a diagnosis other than CP. Three patients in the spine group were excluded, one for incomplete medical records and two for a diagnosis other than CP.

Forty-two patients with CP who underwent hip reconstruction at a mean age of 8.8 years (range 4–21) were compared to 26 patients who underwent PSF at a mean age of 15.4 years (range 10–22). The differences in age were statistically significant (*p* < 0.000001). There were no differences in the GMFCS level between the groups. The patients in the hip group had a mean Reimer’s index of 68 % (range 40–100 %). The patients in the spine group had a mean preoperative Cobb angle of 78° (range 50–120). The overall demographics of each group are detailed in Table 1.

**Fig. 1:**
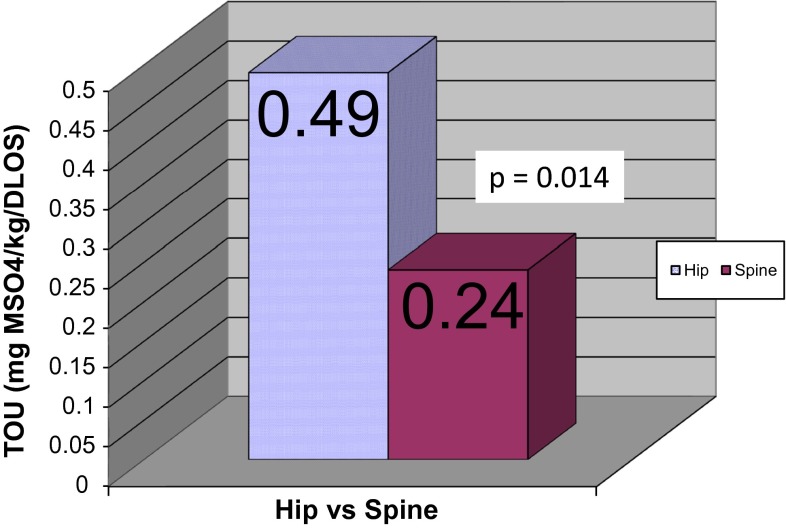
Total opioid used (normalized by body weight and days length of stay [DLOS])

The primary end point of the study, the amount of opioid used, was significantly higher in the hip group when compared to the spine group. In the hip group, the TOU was 0.49 mg morphine/kg/DLOS (range 0.02–2.32), compared to 0.24 for the spine group (range 0.06–0.62) (*p* = 0.014) (Fig. 1).

**Fig. 2:**
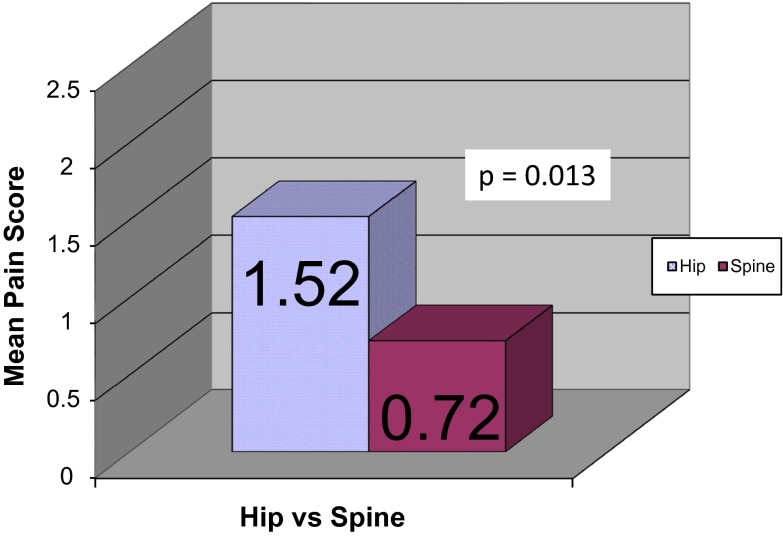
Pain scores for patients undergoing posterior spinal fusion (PSF) or hip reconstruction

The secondary end point of the study, the mean pain score, was also significantly lower in the spine group compared to the hip group. The mean pain score for the hip group was 1.52 (range 0.01–7.00), compared to 0.72 (range 0.0–2.29) for the spine group (*p* = 0.013) (Fig. 2).

There were no statistically significant differences in the occurrence of adverse effects related to pain management between the two groups. Furthermore, subgroup analysis of the hip group showed no differences in TOU or pain scores whether the patient was immobilized postoperatively with a spica cast or with an abduction pillow.

## Discussion

Pain assessment and treatment in the CP population is challenging for both medical providers and families. For many patients, the lack of verbal skills makes it difficult for caregivers to distinguish pain from other sources of discomfort [8]. This patient population is especially vulnerable if family members or guardians are not present to help the staff with patient communication and emotional support [15]. Discussions revolving around pain assessment and treatment are common in dedicated orthopedic clinics [23]. Even with careful evaluation and optimal family support, these issues can be difficult and frustrating.

We chose to compare hip reconstruction with spine fusion for several reasons. First, these are very common operations that patients with GMFCS levels IV and V CP undergo. Secondly, families frequently note the significant pain that hip reconstruction in the form of femoral and/or pelvic osteotomy entails, many telling us that this was the most painful procedure that their child has had to undergo. Similarly, because spine fusion can be such a significant medical event, it is natural that some families assume that spine fusion MUST be at least as painful (if not more painful) than hip reconstruction. Because of that, many families delay the spine fusion significantly. We designed this study to evaluate the pain that patients experience with spine fusion, relative to the amount of pain experienced with hip reconstruction.

In this retrospective series, patients with CP undergoing hip reconstruction surgery had significantly more pain, as exhibited by requiring more narcotics and having higher pain scores, than those patients undergoing PSF. These findings confirm the subjective opinions of many providers that care for these patients, as well as the observations of many patients and their families who have undergone both procedures. The results of this study are not surprising; immobilization generally leads to better pain control, and the immobilization from the spine fusion may be one significant cause in the difference in pain generation when compared to the mobility of a hip that has undergone osteotomy and reconstruction.

While recent reports have focused on the difficulty in assessing pain in patients with CP, we feel that the comparisons made in this study are valid [6, 26]. While as a group the patients with CP may present difficulties in assessing postoperative pain adequately, the two groups in our analysis were very homogeneous [24]. There were no significant differences in GMFCS level, and the patients were treated at the same institution during the same time period. Furthermore, for the purposes of this study, all the patients included had adequate pain assessments.

The focus on family-centered care is shifting our treatment priorities away from isolated clinical outcomes, such as postoperative radiograph findings, and focusing more on patient-centric outcomes. Certainly, the ability to provide adequate postoperative pain control is central to many patients’ and families’ concerns when considering a surgical procedure [16, 20]. A better understanding of the magnitude and the quality of the pain these patients experience after our surgical procedures would better prepare the patients and their families [22]. Also, the realities of modern healthcare are shifting towards a higher focus on patient satisfaction [17]. Indeed, in the future, reimbursement and credentialing may be directly tied to our patients’ satisfaction scores [21]. A better understanding of what to expect postoperatively has been shown to lead to better patient satisfaction ratings [18, 19].

In addition to better patient and parent education, we feel that another very real, practical benefit may come from the findings in these studies. Most hip reconstructions for patients with CP are typically done at an earlier age when compared to the age for PSF, as is shown in our mean age data for each group. A common complaint we hear from our families is how difficult the postoperative period after hip reconstruction can be. For those patients who originally need hip surgery, the authors have observed hesitancy for those families to agree to undergo PSF in a timely fashion. These families often point to the severe postoperative pain after the hip surgery for their delay in seeking treatment for the scoliosis, thinking the spine surgery must be “at least as painful” as the hip surgery. In our experience, this delay has caused some children’s scoliosis to progress severely, thus making the ultimate surgical treatment more difficult, with a concordant higher risk of postoperative complications.

Our study has several limitations. The retrospective nature of the review lends the study to be subject to a variety of bias-related errors. Secondly, the previously mentioned difficulty in assessing and treating postoperative pain in patients with CP could be a significant confounding variable [26]. Thirdly, a single pain score may not fully represent the overall pain management for a patient over a postoperative course. We chose to use a single, averaged score to make a more simplified comparison between the two groups. By using an average pain score, our reported scores in this paper were relatively low, because at many time points, the clinical pain score was zero: the preferred patient outcome when measuring pain. There were significantly high pain scores at times during the course of treatment in each group; the differences in the mean values demonstrate that the overall pain control was better, with lower pain scores, in the spine group compared to the hip group. Finally, our results only include patients from our institution; however, we believe that our deficiencies with pain management in CP patients occur commonly in many centers that treat these patients.

In conclusion, patients with CP undergoing hip reconstruction surgery had significantly more pain, as exhibited by requiring more narcotics and having higher pain scores, than those patients undergoing PSF. The knowledge that hip reconstruction is more painful than PSF for patients with CP will better prepare families about what to expect in the postoperative period and will alert providers to provide better postoperative pain control in these patients.

## References

[1] McKearnan KA, Kieckhefer GM, Engel JM, Jensen MP, Labyak S (2004). Pain in children with cerebral palsy: a review. J Neurosci Nurs.

[2] Dzienkowski RC, Smith KK, Dillow KA, Yucha CB (1996). Cerebral palsy: a comprehensive review. Nurse Pract.

[3] Fanurik D, Koh JL, Harrison RD, Conrad TM, Tomerlin C (1998). Pain assessment in children with cognitive impairment. An exploration of self-report skills. Clin Nurse Res.

[4] Roscigno CI (2002). Addressing spasticity-related pain in children with spastic cerebral palsy. J Neurosci Nurs.

[5] Nolan J, Chalkiadis GA, Low J, Olesch CA, Brown TC (2000). Anaesthesia and pain management in cerebral palsy. Anaesthesia.

[6] Collignon P, Giusiano B (2001). Validation of a pain evaluation scale for patients with severe cerebral palsy. Eur J Pain.

[7] Russo RN, Miller MD, Haan E, Cameron ID, Crotty M (2008). Pain characteristics and their association with quality of life and self-concept in children with hemiplegic cerebral palsy identified from a population register. Clin J Pain.

[8] Breau LM, Camfield CS, McGrath PJ, Finley GA (2003). The incidence of pain in children with severe cognitive impairments. Arch Pediatr Adolesc Med.

[9] Oberlander TF, O’Donnell ME, Montgomery CJ (1999). Pain in children with significant neurological impairment. J Dev Behav Pediatr.

[10] McGrath PA (1990). Pain in children: nature, assessment, and treatment.

[11] Russman BS (2000). Cerebral palsy. Curr Treat Options Neurol.

[12] Liptak GS, O’Donnell M, Conaway M (2001). Health status of children with moderate to severe cerebral palsy. Dev Med Child Neurol.

[13] Gajdosik CG, Cicirello N (2001). Secondary conditions of the musculoskeletal system in adolescents and adults with cerebral palsy. Phys Occup Ther Pediatr.

[14] Hemsley B, Balandin S, Togher L (2008). Professionals’ views on the roles and needs of family carers of adults with cerebral palsy and complex communication needs in hospital. J Intellect Dev Disabil.

[15] Tervo RC, Symons F, Stout J, Novacheck T (2006). Parental report of pain and associated limitations in ambulatory children with cerebral palsy. Arch Phys Med Rehabil.

[16] Capjon H, Bjørk IT (2010). Ambulant children with spastic cerebral palsy and their parents’ perceptions and expectations prior to multilevel surgery. Dev Neurorehabil.

[17] Shirley ED, Sanders JO (2013). Patient satisfaction: implications and predictors of success. J Bone Joint Surg Am.

[18] Lutz GK, Butzlaff ME, Atlas SJ, Keller RB, Singer DE, Deyo RA (1999). The relation between expectations and outcomes in surgery for sciatica. J Gen Intern Med.

[19] O’Holleran JD, Kocher MS, Horan MP, Briggs KK, Hawkins RJ (2005). Determinants of patient satisfaction with outcome after rotator cuff surgery. J Bone Joint Surg Am.

[20] McGregor AH, Hughes SP (2002). The evaluation of the surgical management of nerve root compression in patients with low back pain: Part 2: patient expectations and satisfaction. Spine (Phila Pa 1976).

[21] Jackson JL, Chamberlin J, Kroenke K (2001). Predictors of patient satisfaction. Soc Sci Med.

[22] Anderson R, Barbara A, Feldman S (2007). What patients want: a content analysis of key qualities that influence patient satisfaction. J Med Pract Manage.

[23] Nielsen DM, Gill K, Ricketts DM (2005). Satisfaction levels in orthopaedic out-patients. Ann R Coll Surg Engl.

[24] Merkel SI, Voepel-Lewis T, Shayevitz JR, Malviya S (1997). The FLACC: a behavioral scale for scoring postoperative pain in young children. Pediatr Nurs.

[25] Malviya S, Voepel-Lewis T, Burke C, Merkel S, Tait AR (2006). The revised FLACC observational pain tool: improved reliability and validity for pain assessment in children with cognitive impairment. Paediatr Anaesth.

[26] Shrader MW, Jones JS, Falk M, White GW, Segal LS (2012) Concerns about assessment of postoperative pain in children with cerebral palsy undergoing orthopedic surgery: are we undertreating pain in these children? Podium Presentation, Annual Meeting of the Pediatric Orthopedic Society of North America (POSNA), Denver, CO, May 2012

